# Diagnosis of Sciatica

**Published:** 1884-12

**Authors:** 


**The Diagnosis of Sciatica.**—A diagnostic point in sciatica is given by De Beurmann which we have never seen alluded to. The patient lying on his back with the muscles of the leg and back relaxed, the affected leg is raised while in complete extension and flexed upon the abdomen. This causes marked pain in the course of the sciatic, especially intense at the sciatic notch, and the movement is resisted. If, then, the limb be lowered, and while the leg is flexed on the thigh the latter is again carried up on the pelvis, no pain will be left. This phenomenon depends on the fact, verified by De Beurmann in experiments on the cadaver, that great tension of the sciatic is exerted by flexion of the thigh when the leg is extended, but almost none when the leg is flexed.

In the diagnosis of sciatica from crural neuralgia, affection of the femur, or coxalgia, in all of which diseases the limb and seat of the pain may be similar, this manœuver is of value. If the nerve trunk is free of disease there will be no difference in the amount of pain caused by the extension or relaxation of the nerve by the different positions indicated. In other words, in affections other than sciatica, the movements given to the coxofemoral articulation will be equally painful whether the leg is extended or flexed on the thigh.—*Boston Med. and Surg. Jour.*

